# Stereochemistry of two pheromonal components of the bumblebee wax moth, *Aphomia sociella*

**DOI:** 10.1038/s41598-020-59069-1

**Published:** 2020-02-07

**Authors:** Erika A. Wallin, Blanka Kalinová, Jiří Kindl, Erik Hedenström, Irena Valterová

**Affiliations:** 1 Mid Sweden University, Department of Chemical Engineering, SE-851 70 Sundsvall, Sweden; 2 Institute of Organic Chemistry and Biochemistry of the Czech Academy of Sciences, Research Group of Infochemicals, 166 10 Prague, Czech Republic; 3 Faculty of Forestry and Wood Sciences, Extemit-K group, Czech University of Life Sciences, 165 21 Prague, Czech Republic; 4 Faculty of Forestry and Wood Sciences, Department of Forest Protection and Entomology, Czech University of Life Sciences, 165 21 Prague, Czech Republic; 5 Faculty of Tropical AgriSciences, Department of Crop Sciences and Agroforestry, Czech University of Life Sciences, 165 21 Prague, Czech Republic

**Keywords:** Zoology, Behavioural ecology

## Abstract

The bumblebee wax moth, *Aphomia sociella*, is a parasite of bumblebees. In this species, males produce sexual pheromone to attract females, while females produce an aphrodisiac pheromone that initiates male courtship. Both pheromones contain 6,10,14-trimethylpentadecan-2-one (TMPD-one) and the corresponding alcohol, 6,10,14-trimethylpentadecan-2-ol (TMPD-ol) in sex specific quantities. Male sex pheromone consists of 7 components with TMPD-one as a minor one and traces of TMPD-ol. In female aphrodisiac pheromone, TMPD-ol is the major component, while TMPD-one is present in traces. Here we report on the absolute configuration of TMPD-one in male sex pheromone and TMPD-ol in female aphrodisiac pheromone of *A. sociella*. The configuration was determined from GC/MS of prepared (*S*)-acetoxypropionyl esters of TMPD-ol. TMPD-one was first reduced to the alcohol that was then derivatized with (*S*)-acetoxypropionyl chloride. The GC/MS data of obtained diastereoisomers were compared with synthetic standards. The absolute configuration of TMPD-one in the male pheromone was (6*R*,10*R*). The configuration of TMPD-ol in the female pheromone was (2*R*,6*R*,10*R*). Electrophysiological experiments showed that TMPD-one and TMPD-ol are perceived by both sexes. The synthetic standards of naturally produced stereoisomers elicited higher responses than mixtures of all stereoisomers.

## Introduction

The stereochemistry plays an important role in the pheromone communication^1^. Therefore, it is of great importance to determine the stereoisomeric composition of pheromonal components. *Aphomia sociella L*. (Pyralidae, Galleriinae) is a parasite of bumblebees. Unlike most Lepidoptera, the reproductive behavior of *A. sociella* is initiated by males and except chemical communication (sex pheromone) it includes also an ultrasonic part. The male sex pheromone attracts females from a distance, while ultrasonic signaling urges females to accept mating^2^. Females produce aphrodisiac pheromone, which initiates male courtship behavior^3^. Male pheromone is a multicomponent blend consisting of (*Z*)-nona-2,6-dien-4-olide and mellein as major components, and 1-hexanol, 2-phenylethanol, (*Z*)-nona-6-en-4-olide, and 6,10,14-trimethylpentadecan-2-one (TMPD-one, trivial names phytone or hexahydrofarnesylacetone) as minor components^4,5^. In female aphrodisiac pheromone, 6,10,14-trimethylpentadecan-2-ol (TMPD-ol) represents the major component with TMPD-one and hexan-1-ol as minor ones^3^.

TMPD-one has been found earlier within male sex pheromone of other Galleriinae, such as *Galleria mellonella*^6^ and *Tirathaba mundella*^7^. TMPD-ol plays a role in the communication of another species of this family, *Corcyra cephalonica*, where females utilize TMPD-ol for attracting males^8^. Both TMPD-one and TMPD-ol are chiral compounds, but in none Galleriinae species, the absolute configuration has been reported.

The stereochemistry of these two compounds was determined in other insects beside Galleriinae. TMPD-ol in *Bicyclus* butterflies has been determined after (*S*)-2-acetoxypropionyl derivatization and the formed diastereomers were separated by gas chromatography (GC)^9,10^. The stereochemistry of TMPD-one found in *Euglossa* bees was reported by Eltz^11^, where reduction of ketone was done prior to derivatization. This method was also used to determine the stereochemistry of TMPD-one extracted from *Bicyclus* butterflies^10^. Here we present the stereochemistry of TMPD-one in male sex pheromone and TMPD-ol in female aphrodisiac pheromone of *A. sociella* as well as the electroantennographic activity of these compounds. In addition, we also show for the first time that males produce minute amount of TMPD-ol and that female antennae respond to this compound.

## Materials and Methods

### Insects

Laboratory-started nests of buff-tailed bumblebee (*Bombus terrestris*) were obtained from The Research Institute of Fodder Crops (Troubsko, Czech Republic)^12^. The nests were exposed to colonization by *A. sociella* L. (Pyralidae, Galleriinae) in the field (Prague area). In June/July, the bumblebee hives were situated in the garden and let parasitize by natural population of *A. sociella*. In autumn, the infested hives with larvae were transferred to refrigerator and kept 1 month at 5 °C for the larvae to undergo hibernation. After hibernation, the hives were kept in the rearing room (24 °C, 40% relative humidity). Larvae were allowed to develop and pupate in the bumblebee hives. The newly emerged adults were collected and sexed. The sexes were kept separately first in continuous light under ambient laboratory temperature (20–23 °C). Adults (1–2 days old) were transferred to the dark and used for pheromone extraction. Moths (one day post-emergence) for electrophysiological experiments were kept in a refrigerator with the temperature set to 5 °C and in a high-humidity environment (90%) until used.

### Sample preparation

#### Male sex pheromone

The pheromone is produced in wing glands that are located on the basal part of the forewing, analogically as in *Eldana saccharina* Walker (Lepidoptera, Galleriinae) published by Farine^13^. Glands of 20 calling males were clipped out, pooled together and soaked in redistilled *n*-hexane (Merck, 50 μL per gland). Samples were sonicated for 15 min, and extracted for 24 h. The extracts were filtered through purified glass wool, concentrated under argon flow to double the initial concentration, and stored at −20 °C.

#### Female aphrodisiac pheromone

Virgin females (1–2 days old) were placed individually in cages, and positioned next to caged calling males. Females (20 specimens) that elicited calling songs in males were cooled to −20 °C for 15 min, and their whole bodies (without wings) were pooled and extracted with *n*-hexane (500 μL *n*-hexane per female). After 24 h, the female bodies were removed from the extract, the solution was filtered through the glass wool, concentrated to 2 mL under the stream of argon, and the samples were stored at −20 °C.

### Analysis of crude extract

Extracts from males and females were analyzed using a Hewlett-Packard 6890 N gas chromatograph (GC) equipped with a polar Varian FactorFOUR VF-23ms column (30 m × 0.25 mm i.d., d_f_ = 0.25 µm) and a HP 5973 MS-detector in electron ionization mode (EI). The carrier gas (1 mL min^−1^) was helium; the sample (1 µL) was injected in the splitless mode, the injector temperature was 250 °C, the transfer line was maintained at 250 °C and the MS source was set to 280 °C. For identification of the alcohol and ketone in crude extracts, the full scan mode was used and the column temperature increased from 50 °C by 10 °C min^−1^ up to 230 °C and held at 230 °C for 10 minutes. Mass spectra of main extract components are in the Supplementary material (Figs. S1–S3).

### Purification of insect extracts

Extracts were purified using liquid chromatography (LC). Prior purification, female extracts were washed with NaHCO_3_ (sat.aq.) to remove free carboxylic acids. Afterwards, male and female extracts were treated in the same way. In LC purification, pre-conditioned solid phase extraction column (500 mg, SPE, Chromabond) were used. SPE conditioning was done with EtOAc (1.5 mL), 10% EtOAc in pentane (1.5 mL) and pentane (1.5 mL). Then the columns were gradually eluted in steps of 1% increase of ethyl acetate in pentane (0–15%) with each fraction consisted of a volume of 750 µL.

**Figure 1:**
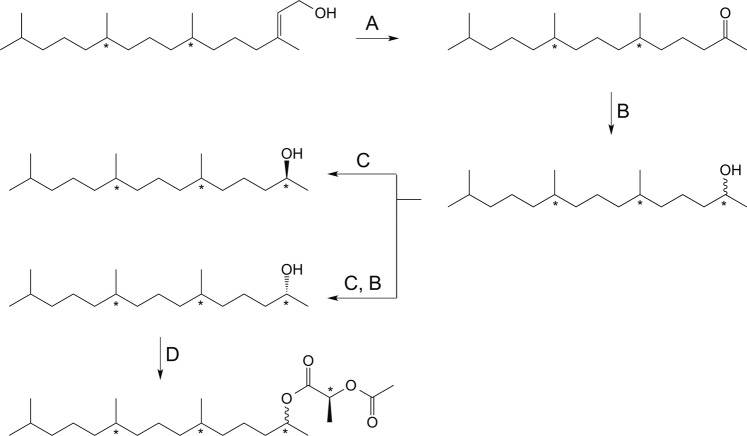
Synthesis of reference compounds and derivatization of reference compounds and natural compounds enriched from insects extracts. (**A**) NaIO_4_, RuCl_3_(cat.), acetonitrile, water, room temperature. (**B**) Lithium aluminum hydride in diethyl ether, 0 °C 1 h, (**C**) *Candida antarctica* lipase B (CAL-B), 4 A molecular sieves, vinyl acetate, heptane, (**D**) (*S*)-2-acetoxypropionyl chloride, pyridine, dichloromethane and cyclohexane, 80 °C 1 h then HCl (1 M) and pentane/cyclohexane and drying over Na_2_SO_4_ (anhydr.).

TMPD-one was enriched in fractions 3–6 and TMPD-ol was enriched in fractions 9−11. In cases where the purity was not satisfactory, the combined fractions were subjected to the purification protocol again until no further improvement of purity was obtained. The combined TMPD-one fractions were then reduced to alcohols (described below). After reduction, the TMPD-ol fractions and those containing the reduced TMPD-one were derivatized and analyzed as described below (Fig. 1D).

### Reduction of TMPD-one fraction

The collected ketone fractions were pooled and solvent evaporated under a stream of argon. The purified ketone was diluted in dry diethyl ether (1 mL) and 0.2 mL was reduced to alcohol with lithiumaluminumhydride in diethyl ether (1 M, 50 µL) at 0 °C for 1 hour (Fig. 1B). The reaction was quenched by addition of HCl (2 M, 100 µL) and H_2_O (100 µL). Phases were separated and the aqueous layer was extracted with Et_2_O (3 × 200 µL), the combined organic layers was washed with HCl (2 M, 100 µL) and brine (100 µL) and dried over Na_2_SO_4_ (anhydr.) in a Pasteur pipette. The solvent was evaporated under a stream of argon. The sample was diluted with pentane (50 µL) and purified with the same SPE purification method as the crude extracts, prior to derivatization.

### Derivatization

To determine the stereochemistry of TMPD-ol enriched from female extract and TMPD-one enriched from male extract of *A. sociella*, both the pooled alcohol fraction and the reduced ketone fraction were derivatized with (*S*)−2-acetoxypropionyl chloride (SigmaAldrich) according to Bång^14^. Pooled extracted TMPD-ol and reduced TMPD-one were concentrated by means of evaporating solvent under a stream of argon. Dry cyclohexane (50 µL) was added to the vial containing the purified extract and subsequently was 1% pyridine in cyclohexane (65 µL) and 1% (*S*)-2-acetoxypropionyl chloride in dichloromethane (100 µL) added, the resulting mixture was heated at 80 °C for 30–60 minutes under argon atmosphere (Fig. 1D). A needle was inserted into the septum of the vial and the solvent was allowed to evaporate before allowing the vial to reach room temperature. HCl (1 M, 200 µL) and pentane or cyclohexane (1 mL) was added, the organic layer was subsequently dried over Na_2_SO_4_ (anhydr.) in a Pasteur pipette. Solvent was either reduced to approximately 50 μL and the sample was analyzed as described below or the solvent was evaporated completely and sample dissolved in cyclohexane before analysis.

### Stereochemical analysis

The derivatized TMPD-ol was analyzed using GC/MS as described above for the crude extracts. The temperature program was adjusted, starting at 50 °C, increased to 110 °C at a rate of 10 °C min^−1^, further from 110 °C by 0.01 °C min^−1^ up to 115 °C, and from 115 °C by 10 °C min^−1^ up to 230 °C, and held at 230 °C for 10 minutes. Selected ion monitoring (SIM) (*m*/*z* 105, 115, 133, 210, 252) was used for identification of the (*S*)-2-acetoxypropionyl derivatives^10^.

### Preparation of reference compounds

(2*R/S*,6*R/S*,10*R/S*)*-*6,10,14-Trimethylpentadecan-2-ol (2*E*,7 *R/S*,11 *R/S*)-(3,7,11,15)-Tetramethyl-2-hexadecen-1-ol ((2*E*,7*R/S*,11*R/S*)*-*phytol) (SigmaAldrich) was oxidized at room temperature by sodium periodate and catalytic amount of ruthenium (III) chloride (Fig. 1A), the obtained ketone was reduced with LiAlH_4_ in diethyl ether resulting in a mixture of eight isomers of TMPD-ol (Fig. 1B) according to the supplemental material of Nieberding *et al*.^9^. The synthetic mixture was derivatized and analyzed according to the same method as the purified extracts.

(2*S*,6*R*,10*R*)*-*6,10,14-Trimethylpentadecan-2-ol and (2*R*,6*R*,10*R*)*-*6,10,14-trimethylpentadecan-2-ol (2*E*,7*R*,11*R*)-Phytol (TCI America) was treated as described above, resulting in a mixture of (2*R*,6*R*,10*R*)- and (2*S*,6*R*,10*R*)-TMPD-ol (Fig. 1B). The isomeric mixture was subjected to enzymatic resolution employing *Candida antarctica* lipase-B (CALB, Roche Diagnostics batch 90750729) as catalyst and vinylacetate (SigmaAldrich) as acyl-donor (Fig. 1C). With this method the (2 *R*)-alcohol is acetylated and therefore the two isomers are easily separated by means of liquid chromatography. The obtained (2 *R*)-acetate was reduced with litiumaluminum hydride in diethyl ether (Fig. 1B). A diastereomeric purity of 99.8% for (−)-(2*R*,6*R*,10*R*)-TMPD-ol and 97.8% diastereomeric purity of (+)-(2*S*,6*R*,10*R*)-TMPD-ol was obtained as described in the supplemental material of Nieberding *et al*.^15^. The stereoisomerically pure isomers were derivatized and analyzed according to the same method as the purified extracts.

### Electroantennography (EAG)

Detailed description of EAG measurements on *A. sociella* is available in Kalinová *et al*.^5^. Briefly, isolated antennae, glass Ag/AgCl electrodes and Syntech EAG recording and stimulus delivery system were used. Male and female antennal responses to stimulation by 10, 100, and 250 ng of racemic mixtures and pure enantiomers of TMPD-one and TMPD-ol were investigated. Tested compounds were diluted in *n*-hexane. Aliquots of 10 μL of respective solutions were loaded onto the filter paper discs within Pasteur pipette odor cartridges. During stimulation, 0.5 mL of humidized air was flown through loaded odor cartridge and injected into the air stream directed onto the antennal preparation. For each sex and stimulus type, 6 replicates were performed.

The antennal responses from each series of stimulations were normalized to the stimulations by 10 ng of 1-hexenol using the Syntech software and expressed as percents of 1-hexenol responses. For statistical evaluation, the data were subjected to logarithmic transformation to correct for the heteroscedasticity inherent to EAG data. The resulting datasets complied with the assumption of equal variances (tested using Levene’s test) and the means were compared using ANOVA followed by Least Squares Differences post-hoc multiple comparison test, performed in Statistica 8.0.

### Dedication

Dedicated to the memory of Prof. Kenji Mori and his significant contribution to the field of chemical ecology.

## Results and Discussion

### Purification of extracts, derivatization, and analysis

GC/MS has been previously used to determine the stereoisomeric composition of TMPD-ol and TMPD-one in wing extracts of *Bicyclus* butterfly species^10^, stereoisomeric composition of TMPD-one in *Euglossa* bees^11^ and of 3,7-dimethylpentadecan-2-ol in female extracts of pine sawflies^14^, where the stereoisomers of the chiral alcohols were separated as either (*R*)-*trans*-chrysanthemoyl or (*S*)-2-acetoxypropionyl esters. Here we used (*S*)-2-acetoxypropionyl derivatization method, because it is robust and more suitable for derivatization of compounds present at low concentrations^10,14^.

Male gland extracts gave stronger samples than female body extracts obtained from one specimen (Fig. S4). TMPD-one and TMPD-ol were found in both sexes, but their ratio in the sexes differed. In males, the ketone was present in substantially higher amount than the alcohol while in females, the alcohol dominated over the ketone. In the male wing glands, the trace amount of TMPD-ol was on the detection limit and the compound was only found after pre-cleaning the extract with SPE. A rough estimate of TMPD-ol quantity produced by females and TMPD-one produced by males was done using dodecanol as internal standard. In average, males produce 2−7 ng TMPD-one and females produce 1−5 ng TMPD-ol per individual.

**Figure 2:**
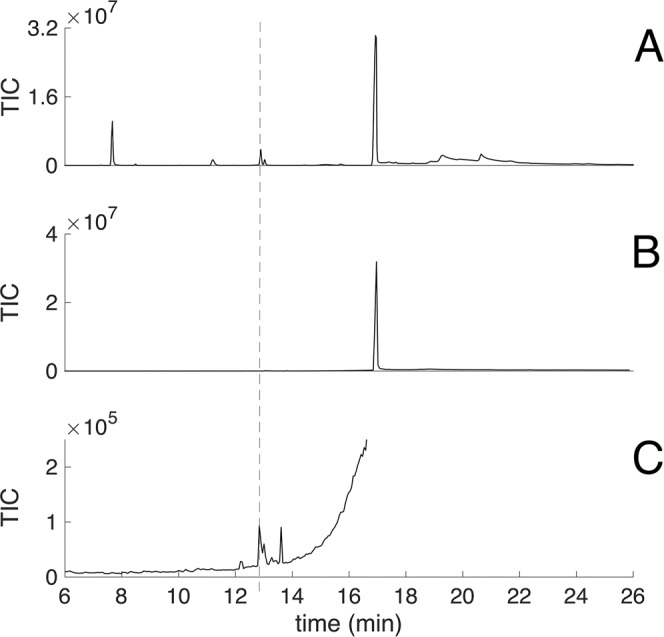
Gas chromatogram of crude male extract, purified male TMPD-ol which is co-eluting with mellein during SPE purification. Dotted line indicates the retention time for TMPD-ol/TMPD-one. (**A**) Crude male extract. (**B**) Full chromatogram of fraction containing TMPD-ol and mellein. (**C**) Chromatogram B zoomed in on y-axis to show the presence of TMPD-ol in collected fractions.

Obtaining pure TMPD-ol from male glands was complicated due to its co-elution with mellein, which is a major component of the male-produced pheromone (Fig. 2A, crude male extract, main peak). In the GC analysis of crude extracts TMPD-ol and TMPD-one co-elute, as a result of this a relatively large peak can be seen at the retention time of TMPD-ol/TMPD-one in the crude extract (Fig. 2A, dotted line). After the first run of purification, mellein still dominated the fraction containing TMPD-ol (Fig. 2B, fraction containing TMPD-ol and mellein; Fig. 2C, fraction containing TMPD-ol and mellein, enlarged in Y-axis). Based on integration, the peak area of TMPD-ol in the fraction was 0.024% of the mellein peak area. The attempt to remove mellein by the reaction with KOH (1 M) was not successful and did not lead to the determination of the configuration of the minute amount of derivatized TMPD-ol.

Similar problems occurred during the purification of the female extract where TMPD-ol dominates over TMPD-one. Since the source of female pheromone is not known^3^, extract of whole female bodies was used. Purification of TMPD-one and TMPD-ol from the females was more difficult due to a higher contamination resulting from body extraction compared to gland extraction in males. TMPD-one from the extract of females was separated from TMPD-ol by means of SPE purification, but unfortunately the fractions were not pure. Several attempts of purification were performed. The ketone fraction was treated with KOH (1 M) in order to remove the excess of free fatty acids; this was however not successful. The ketone fraction was subjected to reduction and derivatization even though there were impurities present; it did not result in unambiguous determination of the stereochemistry. Thus, we were able to determine the stereochemistry of those pheromonal components present in higher amounts, i.e. of TMPD-one in the males and TMPD-ol in the females.

**Figure 3:**
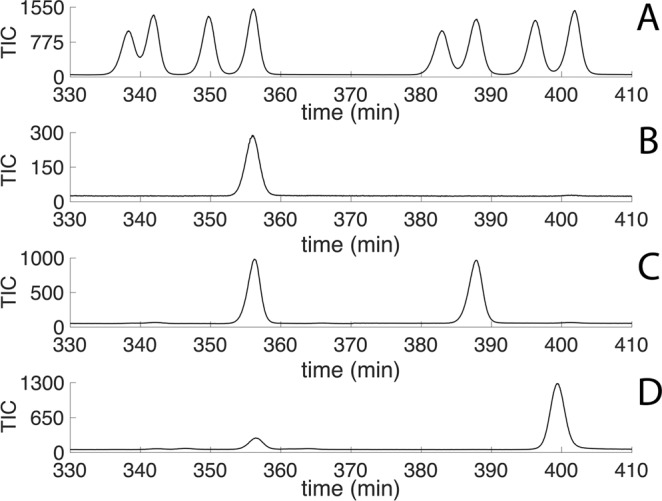
Gas chromatogram showing separation of (**A**) Synthesized and derivatized synthetic eight mixture of (2 *R*/*S*,6 *R*/*S*,10 *R*/*S*)-TMPD-ol. (**B**) Synthesized and derivatized synthetic (2*R*,6*R*,10*R*)-TMPD-ol. (**C**) Reduced and derivatized male-produced TMPD-one. (**D**) Derivatized female-produced TMPD-ol.

The synthetic mixture of eight stereoisomers and pure isomers of (2*R*,6*R*,10*R*)- and (2*S*,6*R*,10*R*)-TMPD-ol were utilized for comparisons on GC/MS. The pure isomers were synthesized by Hedenström^10^ while working with *Euglossa* bees and *Bicyclus* butterflies^10,11^. The eight-stereoisomers mixture of TMPD-ol was synthesized according to Nieberding (supplemental material)^9^. All 8 derivatized stereoisomers separated well as (*S*)-2-acetoxypropionyl esters on the highly polar VF-23ms column (Fig. 3A). The male-extracted, reduced and derivatized pheromonal component, matched with the authentic standard of derivatized (2*R*,6*R*,10*R*)-TMPD-ol (Fig. 3B) leading to a conclusion that males produce (6*R*,10*R*)-TMPD-one (Fig. 3C). During reduction of TMPD-one a new stereocenter is introduced in position 2, that is the reason to why two peaks appear in the chromatogram after derivatization (Fig. 3C). Due to co-elution during SPE purification of the male-produced TMPD-ol with mellein (Fig. 2) however, it was not possible to determine the stereochemistry of the native male TMPD-ol present in male wing glands in minute quantity. Female-produced TMPD-ol was determined as (2*R*,6*R*,10*R*)-TMPD-ol based on the match of the extracted and derivatized compound with the synthetic standard. The GC/MS analysis of female-produced and derivatized TMPD-ol showed one more peak at 397.5 min (Fig. 3D). However, retention time and mass spectral data did not match with any of the TMPD-ol isomers.

Schulz^16^ showed that the Large Cabbage White, *Pieris brassicae*, sequester (*R*,*R*)-phytol from food sources and via oxidative processes transform phytol to (6*R*,10*R*)-TMPD-one. Eltz^11^ reported on *Euglossa* bees carrying (6*R*,10*R*)-TMPD-one in their hind-leg pouch which is believed to be collected from a natural source, despite the source of TMPD-one has not been identified. This shows that some insects utilize the naturally derived (*R*,*R*)-phytol as a precursor. Since we kept *A. sociella* in the old bumblebee hives and not on an artificial diet of known composition, we cannot tell what the source of (2*R*,6*R*,10*R*)-TMD-ol was in this particular case.

The stereoisomers of TMPD-one found in males and TMPD-ol in females of *A. sociella* have the same configuration on chiral centres (2*R*,6*R*,10*R*) as 9 butterfly species of the genus *Bicyclus* studied earlier by Hedenström^10^ and as male orchid bees of the genus *Euglossa*^11^. On the other hand, 3,7-dimethylpentadecan-2-ol, present in the female sex pheromone of the pine sawfly *Neodiprion lecontei*, has the configuration (2 *S*,3 *S*,7 *S*)^14^. The stereochemistry of TMPD-one and TMPD-ol in pheromones of related species of the subfamily Galleriinae (*Galleria mellonella, Tirathaba mundella* or *Corcyra cephalonica*) has not been studied so far. Thus, this paper is the first report on the configuration of the above pheromonal components within Galleriinae.

### Electroantennograhpy

**Figure 4:**
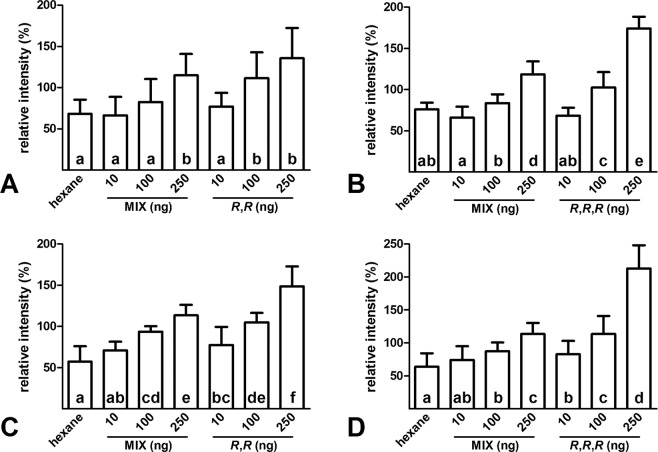
Electroantennographic responses of females (**A,B**) and males (**C,D**) of *Aphomia sociella* to either equimolar mixtures of all stereoisomers or to pure standards of natural isomers (dose-response diagrams). (**A**) Responses of females to the mixture of 4 isomers of TMPD-one compared to its (6*R*,10*R*)*-*isomer. (**B**) Responses of females to the mixture of 8 isomers of TMPD-ol compared to its (2*R*,6*R*,10*R*)*-*isomer. (**C**) Responses of males to the mixture of 4 isomers of TMPD-one compared to its (6*R*,10*R*)*-*isomer. (**D**) Responses of males to the mixture of 8 isomers of TMPD-ol compared to its (2*R*,6*R*,10*R*)*-*isomer. The data were normalized to the stimulations by 10 ng of 1-hexenol and expressed as percents of 1-hexenol responses (mean ± SD).

To determine the biological activity of native stereoisomers of TMPD-one and TMPD-ol, antennal responses of males and females of *A. sociella* were recorded and compared to those of a mixture of eight stereoisomers for TMPD-ol and/or a mixture of four stereoisomers for TMPD-one. Three concentrations for each treatment were tested to see the dose-response dependence (Fig. 4). The lowest selected concentration (10 ng) was approximately of the same order as 2 gland equivalents of males (wing glands are pair organs) or 1 body equivalent of females (the source of pheromone is not known, therefore the extract of the whole body was used). TMPD-one and TMPD-ol were perceived by both sexes. The lowest concentrations of both compounds elicited responses that were not signifficantly different from the solvent (*n*-hexane). Higher doses elicited higher antennal responses that were statistically different from the solvent at the levels 100 ng or 250 ng. Female antennal reactions were higher in response to native stereoisomer of TMPD-one (100 ng) in comparison with TMPD-one stereoisomeric mixture (Fig. 4A), while males responded in the same way on a higher concentration level (250 ng, Fig. 4C). On the other hand, both sexes responded significantly more strongly to the native stereoisomers of TMPD-ol than to the stereoisomeric mixture, both at the concentration level of 100 ng and 250 ng (Fig. 4B,D).

## Conclusion

Based on the purification and derivatization of the extract of male and female pheromone of *A. sociella*, we have determined the absolute configuration of TMPD-one in the male sex pheromone as (6*R*,10*R*)-6,10,14-trimethylpentadecan-2-one and of TMPD-ol in the female aphrodisiac pheromone as (2*R*,6*R*,10*R*)-6,10,14-trimethylpentadecan-2-ol. The pure stereoisomers elicit significantly stronger antennal responses than the tested stereoisomeric mixtures of both TMPD-ol and TMPD-one, respectively. We are aware that in the tested stereoisomeric mixtures only one forth (TMPD-one) or one eights (TMPD-ol) of the naturally produced isomer is present. We are showing here that the antennal responses increased with dose, for both the pure enantiomers as well as the mixtures of all stereoisomers. EAG experiments suggest that the antennal perception may be enantioselective. The significant difference in the perception of the pure isomer and the mixture might mean that none of the non-natural stereoisomers is antennaly active, has synergic affect, or influences the antennal response in any way. However, further experiments with pure stereoisomers are needed to support this hypothesis.

## Supplementary information

Figures S1-S4.
