# Supplementary material for: Stereochemistry of two pheromonal components of the bumblebee wax moth, Aphomia sociella
Source: Sci Rep. 2020 Feb 7;10:2094. doi: 10.1038/s41598-020-59069-1 (PMC7005912; doi:10.1038/s41598-020-59069-1)
Supplement: Supplementary file 1 — Figures S1-S4. [file 41598_2020_59069_MOESM1_ESM.pdf]

Supplementary material, Figures S1-S4. Erika A. Wallin, Blanka Kalinová, Jiří Kindl, Erik Hedenström, Irena Valterová*: Stereochemistry of two pheromonal components of the bumblebee wax moth, *Aphomia sociella*

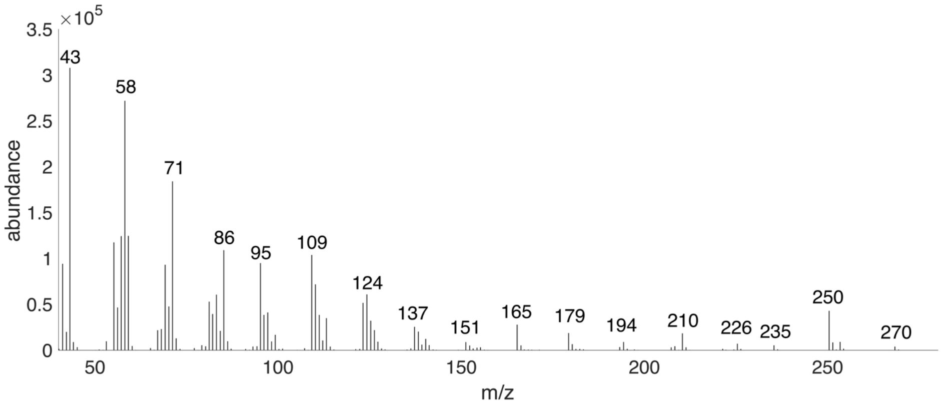

Figure S1. Mass spectrum of TMPD-one.

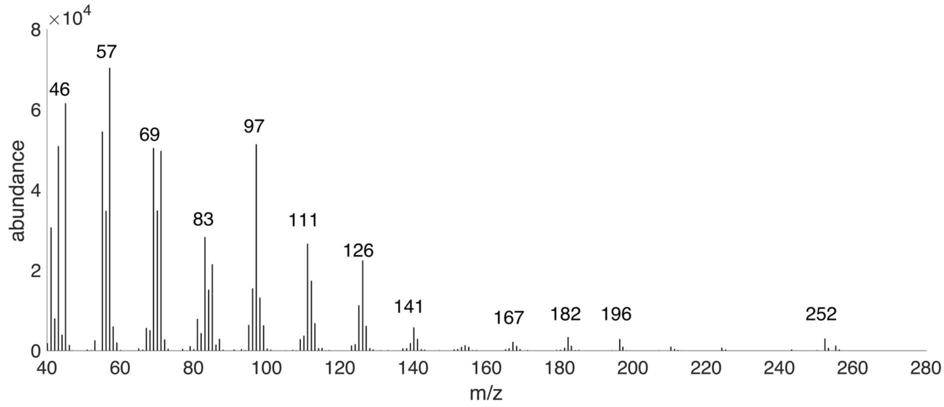

Figure S2. Mass spectrum of TMPD-ol.

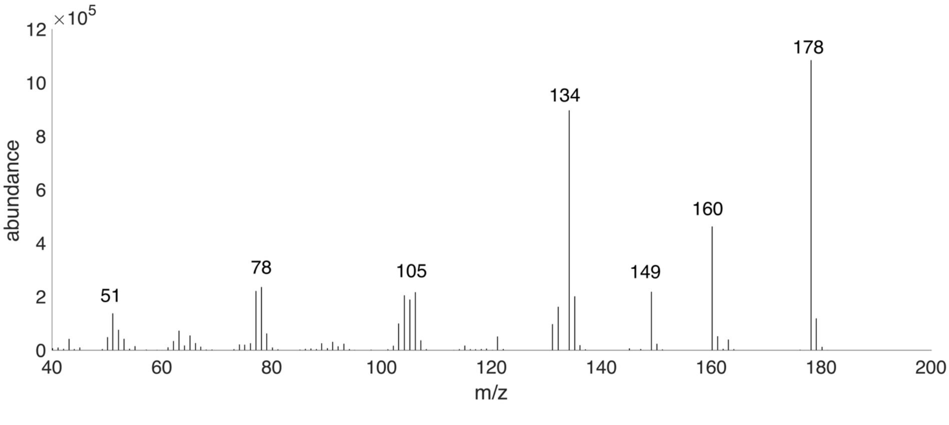

Figure S3. Mass spectrum of mellein.

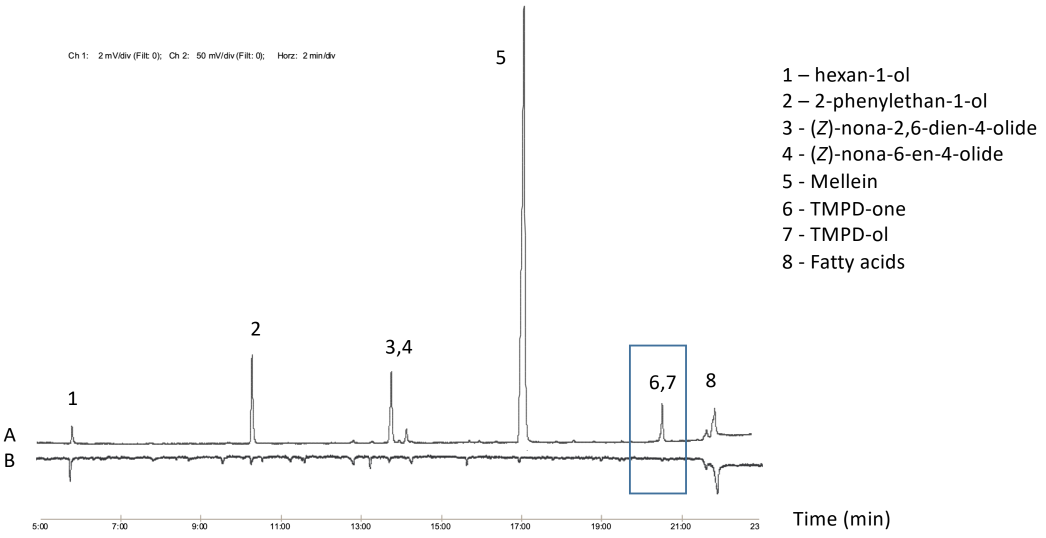

Figure S4. Gas chromatograms of the male wing gland extracts (A, upper trace) and the female body extract (B, lower trace), equivalent of 1 specimen.
